# Dublin Obstetrics

**Published:** 1888-09

**Authors:** E. S. McKee

**Affiliations:** Cincinnati, Ohio


### DUBLIN OBSTETRICS.

By E. S. McKee, M. D., Cincinnati, Ohio.

Ephraim McDowel, born in Dublin, June, 1798, died December, 1835, was a cousin of our own Ephraim McDowell, of Kentucky. That reputation for which the Dublin School of Midwifery is so noted is due largely to the Rotunda and her students, who number now about seven thousand. These are doing good work not only in the British Isles, but also in the Colonies, America, France and Germany, and other countries. The annual number of women delivered at the hospital is about 1,200, and in the extern department about 1,600. Laceration of the perineum, of more than three-quarter inches, is treated at the Rotunda by douching out the vagina with antiseptic solution and suture immediately with silk or cat-gut, using the continuous suture with the latter. The stitches gather and prevent the entrance of lochia into the wound.

Corrosive sublimate for the hands 1.1500, and for intra-uterine injection 1.4000 has almost entirely taken the place of carbolic acid solution. In cases of considerable hemorrhage, suspected renal mischief or great corpulence where absorption is great and mercurial poisoning is fearful, 1.100, or 1.80 solution of carbolic acid is used. Evaporation of carbolic acid in the wards is now discontinued, as is the use of iodoform pessaries, excepting in cases where there is much fetid discharge after first washing out the uterus. The napkins used are soaked in a sublimate solution of 1.500 to 1.000 and wrung out before being applied to the vulva. The mortality from any form of septic infection is fifty-two per cent., or one in 189.6. Eighty-five per cent. of the cases do not exceed the maximum physiological temperature, 38° centigrade or 100.4° Fahrenheit.

Forcep cases are about six per cent. of all cases. Of these there is a mortality of about three per cent. Axis traction forceps are used in a large proportion of cases.

Infantile asphyxia is treated after the method of Schultze. The incubator is used with a considerable degree of success with asphyxiated children, also those whose circulation is bad.

The prophylactic treatment of ophthalmia neonatorum is never adopted till the disease is manifest. Nitrate of silver solution, 8 grains to the ounce, is then applied to both eyes, even though but one is affected. The proportion of these cases is 1.100

Hour-glass contraction is believed to be due to pressure on the ring or band, which causes the circular fibres to contract tonically and may imprison the placenta or a part of it. The difficulty is sometimes removed by douching the uterus with a hot sublimate solution. When the placenta is adherent the hand is passed into the uterus, first being made antiseptic.

Careful external examination is recommended to be made of every pregnant woman a fortnight before the expected confinement and thus determine whether mal-position or conditions favoring it are present.

Membranes retained—gentle traction is made on the part left behind, as a rule, the ligature has been tied on the retained portion as near as possible to the vulva. It is then twisted into a sort of rope whereby its chances of breakage is lessened. If a large amount is left behind the douche is often used in the hope that a portion or all of it may be brought away.

The bimanual is made with the patient lying on her back, contrary to the general practice in Great Britain, and great stress is laid on this position.

External dilatation in the diagnosis of pregnancy receives much merited attention at Dublin. They determine the fact of pregnancy, period, size of the child, condition of the bladder, axis of the uterus, condition of the abdominal walls, abnormalities of the ovaries, such as twins, excessive liquor amnii, hydrocephalus, anencephalus, hydatid mole, and frequently the life or death of the fœtus. It directly leads to the practice of external turning, renders the finding of the fœtal heart simple and often tells whether the pelvis is contracted or not, the amount of disproportion between the head and the pelvis. Such accuracy is attained that they are often able to feel the arm of the child growing out from the shoulder, and to get the fingers in the groin, and occasionally the elbow is palpated. A hydrocephalic head has been readily made out and in one case a anencephalis. In some instances the cord has been felt crossing the child's back and the placental site has been determined by the fingers being lifted off the child and the outlines of the body being rendered indistinct. To make this possible the placenta must be implanted on the anterior wall and the conditions for palpation favorable.

In the third stage of labor, as soon as the child is born, the left hand is placed on the fundus, the ulnar edge pointing towards the vertebral column, with the palm of the hand looking directly in the axis of the uterus. The finger passed in to learn if the cord is around its neck. If the uterus fails to act promptly or the child shows symptoms of defective aeration, pressure is made on the fundus, which is generally sufficient to expel the child. If not, gentle pressure is exerted on the head, which presses it backward toward the perineum till the anterior shoulder passes with a well marked jerk past the pubes, and delivery is terminated by pressure over the fundus uteri. The left hand follows down the fundus as the child leaves the uterus, expelling any liquor amnii which may remain. The head remains in the same position as before the child was born. The accoucher rotates the hand so that the fingers irritate the posterior and lateral sides of the uterus, especially the insertion of the round ligaments. Improperly done, this pressure may do harm instead of good. The fundus may be deflected to one side, generally the left, and if pressure be made in the median line this flexion is still more increased, folding the uterus on itself and pressing towards the fundus rather than towards the os. By means of his hand the attendant keeps himself posted by watching alternate relaxations and contractions as to whether the uterus is becoming widely distended with blood or not. The nurse ties the cord as soon as pulsations have ceased, and the medical man never loses his grip on the uterus. If in a state of asphyxia the physician directs his attention to the child, and the nurse follows the uterus. If there is a tendency to post partum hemorrhage on the part of the mother and asphyxia in the child, the physician must be bound under these delicate circumstances to look after the welfare of the mother. No accumulation of blood taking place in the uterus, gentle friction is used every fifteen minutes, and firm pressure is made on the fundus in the axis of the brim and at the acme of the contraction. This nearly always expels the placenta from the uterus and the vagina. The hand is still kept over the uterus till there is no danger of a recurrence of hemorrhage, when the binder is applied and the woman turned over on her back. This has been the method employed in the Dublin lying-in hospital since the mastership of Dr. Joseph Clark, 1786–1793. In the wards of Dr. Collins, a duty of paramount importance was to press out the placenta on the uterus during the third stage of labor. This method should be called by the name of the city in which it first saw light, the Dublin method.

